# Evaluation of surface roughness of titanium implants on human fibroblast cells

**DOI:** 10.1038/s41598-026-46836-9

**Published:** 2026-06-02

**Authors:** Maleeha Al Hamadani, Arshiya Banu, Karolina Wieczorek, Carlo Seneci, Hassna Irzan, Luis C. Garcia-Peraza-Herrera, Devy F. Garna, Samantha Ya Terry, Lucy Di-Silvio, Prashant Jha, Sebastien Ourselin

**Affiliations:** 1 Research Department of Surgical and Interventional Engineering, School of Biomedical Engineering & Imaging Sciences, King’s College London, Westminster Bridge, SE1 7EH London, UK; 2 Research Department of Imaging Chemistry and Biology, School of Biomedical Engineering & Imaging Sciences, King’s College London, Westminster Bridge, SE1 7EH London, UK; 3 Centre for Oral, Clinical and Translational Sciences, Faculty of Dentistry, Oral & Craniofacial Sciences, King’s College London, Guy’s Hospital, SE1 9RT London, UK

**Keywords:** Biotechnology, Diseases, Materials science, Medical research

## Abstract

A soft-anchored titanium (Ti-6Al-4V) mesh implantable device was designed and morphologically characterised in an *in vitro* study. The mesh implant consists of a titanium mesh aimed at preserving continence and improving the quality of life for patients with a stoma. Titanium alloys are widely used in implantable devices such as knee and dental prosthetics; however, soft-anchored or percutaneous implant options remain limited. Surface roughness is known to influence cell adhesion and proliferation; thus, three titanium samples were produced with different surface finishes: non-polished (NP), matte polished (MaP), and mirror polished (MiP). Comparative analyses were conducted via cell metabolic activity, cytotoxicity, and immunocytochemistry assays with adult normal human dermal fibroblasts (NHDFs). No significant difference in NHDF metabolic activity was observed ($p> 0.8845$), and the cytotoxicity results revealed no toxicity ($p> 0.9999$) between the surfaces. Collagen type I expression was 568.86 ± 88.12, 433.26 ± 147.02, and 681.52 ± 86.14 $\mu m^2$ for NP, MaP, and MiP, respectively, whereas it was 544.54 ± 110.69 $\mu m^2$ in the controls ($p> 0.2193$). Fibronectin-positive areas were 438.24 ± 109.05, 336.97 ± 80.22, and 311.62 ± 88.66 $\mu m^2$ for NP, MaP, and MiP, respectively, while they were 318.82 ± 52.56 $\mu m^2$ for the controls ($p> 0.0507$). The results indicate that surface roughness (Ra) did not significantly affect cell proliferation, cytotoxicity, or ECM protein expression. Thus, under these *in vitro* conditions, we observed no detectable detrimental effect of surface roughness on fibroblast metabolic activity, cytotoxicity, or ECM protein expression, suggesting that extensive polishing steps may not be essential for achieving cytocompatibility of Ti-6Al-4V mesh components in devices designed for stoma patients, although further preclinical studies are required before any clinical recommendations can be made.

## Introduction

The incidence of bowel cancer and various inflammatory bowel diseases (IBDs), such as Crohn’s disease and ulcerative colitis, has increased worldwide. It is estimated that, worldwide, more than 1.90 million people currently live with colorectal cancer, and 4.90 million people live with IBD^1–4^. Stoma surgery, a prevalent solution for these conditions, involves pulling a healthy portion of the bowel through the abdominal wall and attaching it to the skin^1–5^. It is estimated that every year in the UK, there are 21,000 new stoma patients and a total of one million ostomy patients in China and one million ostomy patients in the USA^3,6^. Nonetheless, stoma patients have encountered noticeable physical and psychological challenges from the use of stoma bags, such as leakage, unpleasant odours, frequent bag replacements, skin irritation, bowel infection, and the development of parastomal hernias. All these challenges disrupt their daily routines^7–15^. To increase the quality of the user experience and quality of life for stoma patients, we developed a two-piece implantable device comprising a titanium mesh and a flexible valve.

Implantable devices play a crucial role in the field of medical technology, which is characterised by steady advancements that have significantly improved patient care. These devices, ranging from cardiac pacemakers ensuring steady heart rhythms to neural implants that restore lost functionalities, have introduced transformative changes to healthcare practice^16–20^. In recent years, remarkable progress has been made in implantable device materials, design, and functionality. Consequently, these devices have not only evolved in terms of effectiveness but also become safer and more biocompatible^21–24^. The confluence of biocompatible materials, the capabilities of three-dimensional (3D) printing and advances in computer-aided design (CAD) have paved the way for the creation of increasingly intricate and patient-tailored implantable devices with a cost-effective manufacturing process^25–28^. While various bone-anchored implants, such as hip and knee prosthetics and dental implants, are widely available, the selection of soft-anchored or percutaneous implants remains relatively limited^29–33^. Consequently, it is necessary to annotate the performance and behaviour of implantable devices within the context of the abdominal wall, as its anatomy encompasses several layers, including the skin, subcutaneous fat, deep fascia, abdominal muscles, transversalis fascia, extraperitoneal fat, and parietal peritoneum^34,35^.

One of the main materials used for implantable devices is titanium^36–40^. The advantage of using titanium over other implantable-grade metals lies in its low modulus, high corrosion resistance and fatigue strength, high biocompatibility, and low toxicity^38,41–46^. In addition to having a biocompatible property, the material can be 3D printed via the additive manufacturing (AM) 3D printing technique, which enables the creation of more complex and porous structures that can facilitate cell growth and cell attachment to the implant. Among metal AM routes, direct metal laser sintering (DMLS), a laser powder bed fusion process, typically produces parts with relatively high as-built surface roughness compared with conventionally machined components, which can adversely affect surface quality and often requires additional post-processing. This limitation further motivates systematic evaluation of how such roughness influences soft-tissue cell responses on Ti-6Al-4V meshes^47^. Ti-6Al-4V, one of the most widely used titanium alloys, is employed in joint, dental and cardiovascular implants, such as prosthetic valves, pacemakers and other circulatory devices, owing to the presence of both alpha (Al) and beta (V) stabilisers that enhance the strength of the alloy^48–51^. AM of Ti-6Al-4V has also been extensively reviewed with respect to process parameters, microstructure, defect formation and mechanical behaviour^52–54^.

As titanium implant surfaces are exposed to the surrounding tissues, the behaviour of the cells is determined by the surface of the implant. Recent studies have investigated the effects of different surface topographies on the viability of cells surrounding implants^55–65^. Several studies have demonstrated that treating the surface of titanium via different technologies, including both physical and chemical surface modifications such as UV treatment, metallographic polishing, sandblasting, and various chemical methods for smoothing the surface, leads to better cell adhesion and cell differentiation than does the use of an untreated titanium surface^66–70^. In a study carried out by Fojt *et al*^66^., surface adjustment reduced corrosion and led to improved cell adhesion. Ikeda *et al*^67^. further showed that surface modification through UV treatment enhanced cell adhesion and accelerated the attachment of fibroblasts. Other studies demonstrated that low cell proliferation was linked to greater surface roughness^68^, and illustrated that a smoother surface of Sa less than $0.2\ \mu$m increased human gingival fibroblast attachment^69^. Similarly, decreasing the roughness of the titanium surface improved the spread of fibroblasts, thereby increasing cell proliferation and adhesion^70^.

In contrast, several studies have shown that having a rougher surface increases the surface area, which results in increased cell proliferation and cell adhesion^71,72^. A similar trend was observed by Velasco *et al.*^73^, where surface-modified titanium leading to a rougher surface resulted in enhanced bone contact and growth, and Gehrke *et al.*^74^, where the highest surface roughness samples presented higher bone-implant contact (BIC) and bone area fraction occupancy (BAFO%).

Other studies have reported no difference between the rough surface and smooth surface of titanium, where the different surface treatments compared with a control sample (rough surface) resulted in similar levels of cell adhesion and cell proliferation^75^. To the best of our knowledge, few studies have investigated the cell proliferation and cell adhesion of soft tissues^76–79^.

In this study, we investigated the cellular behaviour of a soft-anchored titanium mesh implant considering different surface modifications and roughnesses, including non-polished (NP) and two differently polished surfaces, namely, matte-polished (MaP) mesh and mirror-polished (MiP) mesh.

## Results

### Surface characterisation analysis of the Ti-6Al-4V

**Fig. 1:**
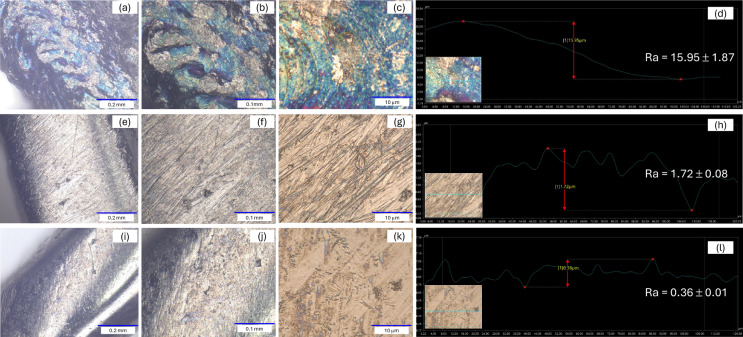
4K-Ultra High Accuracy VHX-7000N Digital Microscope images of the three different surfaces: the top row (a, b, and c) depicts the Non-polished (NP) surface, the middle row (e, f, and g) depicts the Matte-polished (MaP) surface and the bottom row (I, j, and k) depicts the Mirror-polished (MiP) surfaces. The roughness value (Ra) of each surface is measured by the same microscope shown in the last column (d, h, and I).

The surface morphology and Ra values of the Ti-6Al-4V mesh samples before and after polishing were characterised, and the results are presented in Fig. 1. The NP sample exhibited a visibly rough and textured surface typical of as-built additive manufacturing, with a measured Ra of 15.95 ± 1.87 $\mu$*m* (Fig. 1a-d). Following MaP, the surface became smoother and more uniform, with Ra reduced to 1.72 ± 0.08 $\mu$*m* and minor surface irregularities remaining visible (Fig. 1e-h). MiP surface displayed the highest degree of finish, appearing bright and reflective with no noticeable scratches or texture, achieving Ra of 0.36 ± 0.01 $\mu$*m* (Fig. 1i-l). This progressive reduction in surface roughness, spanning nearly two orders of magnitude from NP to MiP, provides a wide morphological range to assess fibroblast responses across different surface finishes, from highly textured as-built to near-smooth engineered surfaces. The quantitative Ra values are summarised in Table 1.

**Table 1:** Surface roughness Ra of the three surfaces, including Non-polished (NP), Matte polished (MaP), and Mirror polished (MiP).

Sample Name	Sample Code	Ra
Non Polished	NP	15.95 ± 1.87 $\mu$m
Matte Polished	MaP	1.72 ± 0.08 $\mu$m
Mirror Polished	MiP	0.36 ± 0.01 $\mu$m

### Cellular metabolic activity

**Fig. 2:**
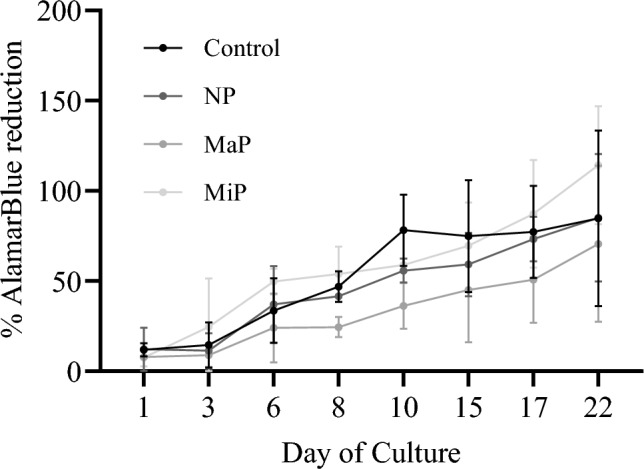
Alamar Blue viability and proliferation Assay for normal human dermal fibroblasts incubated with Non-polished (NP), Matte-polished (MaP), and Mirror-polished (MiP) titanium meshes. N=3.

The metabolic activity and cell proliferation of NHDFs cultured on NP, MaP and MiP Ti-6Al-4V mesh surfaces were assessed, as depicted in Fig. 2. NHDF metabolic activity increased progressively over the 22-day culture period across all groups, indicating ongoing cell proliferation in both control wells and titanium surfaces. On day 1, no significant differences in metabolic activity were observed between the three surface finishes. By day 22, metabolic activity on NP, MaP and MiP surfaces was 85.14 ± 35.31%, 70.61 ± 43.27% and 114.30 ± 32.65% relative to the untreated control, respectively, with no statistically significant differences detected between surfaces ($p> 0.8845$), although substantial variability in the data may limit sensitivity to subtle effects. Notably, despite the wide range in surface roughness (Ra $\approx$ 0.36–15.95 $\mu$m), all three polishing variants supported comparable cell proliferation trajectories, demonstrating that the surface finish did not exert a significant inhibitory or stimulatory effect on NHDF metabolic activity under these culture conditions.

### Cytotoxicity

**Fig. 3:**
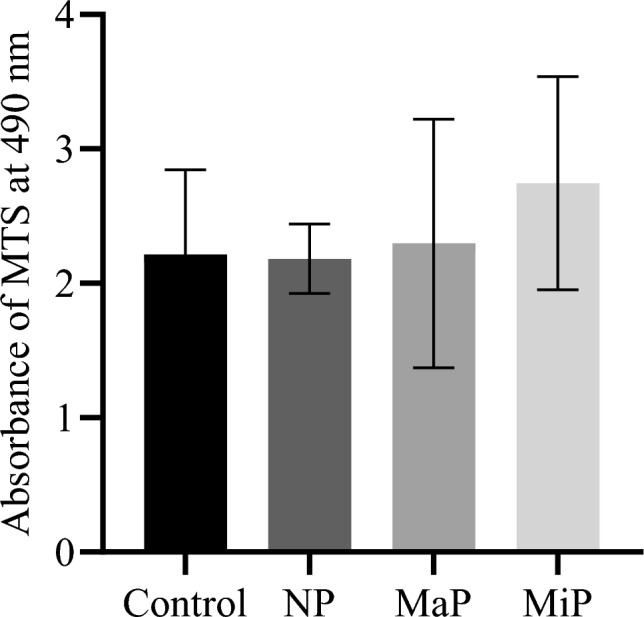
Viability of untreated normal human dermal fibroblasts (NHDF) or NHDF incubated with different titanium meshes, namely Non-polished (NP), Matte-polished (MaP) and Mirror-polished (MiP), at day 5. N=3.

MTS assay results revealed that all three Ti-6Al-4V surface finishes did not elicit cytotoxic effects against cultured NHDFs as depicted in Fig. 3. After 5 days of culture at an absorbance of 490 *nm*, absorbance values for NP, MaP and MiP meshes were 2.18 ± 0.26, 2.29 ± 0.93 and 2.07 ± 0.98, respectively, compared with 2.22 ± 0.63 for untreated control wells. No statistically significant differences in cell viability were detected between any of the three surface finishes or relative to the control ($p> 0.9999$). The results demonstrate that none of the surface morphologies, ranging from highly textured as-built (NP, Ra $\approx$ 15.95 $\mu$*m*) to near-smooth engineered finishes (MiP, Ra $\approx$ 0.36 $\mu$*m*), induced detectable cytotoxic responses in NHDFs.

### Fibronectin and collagen immunocytochemistry

**Fig. 4:**
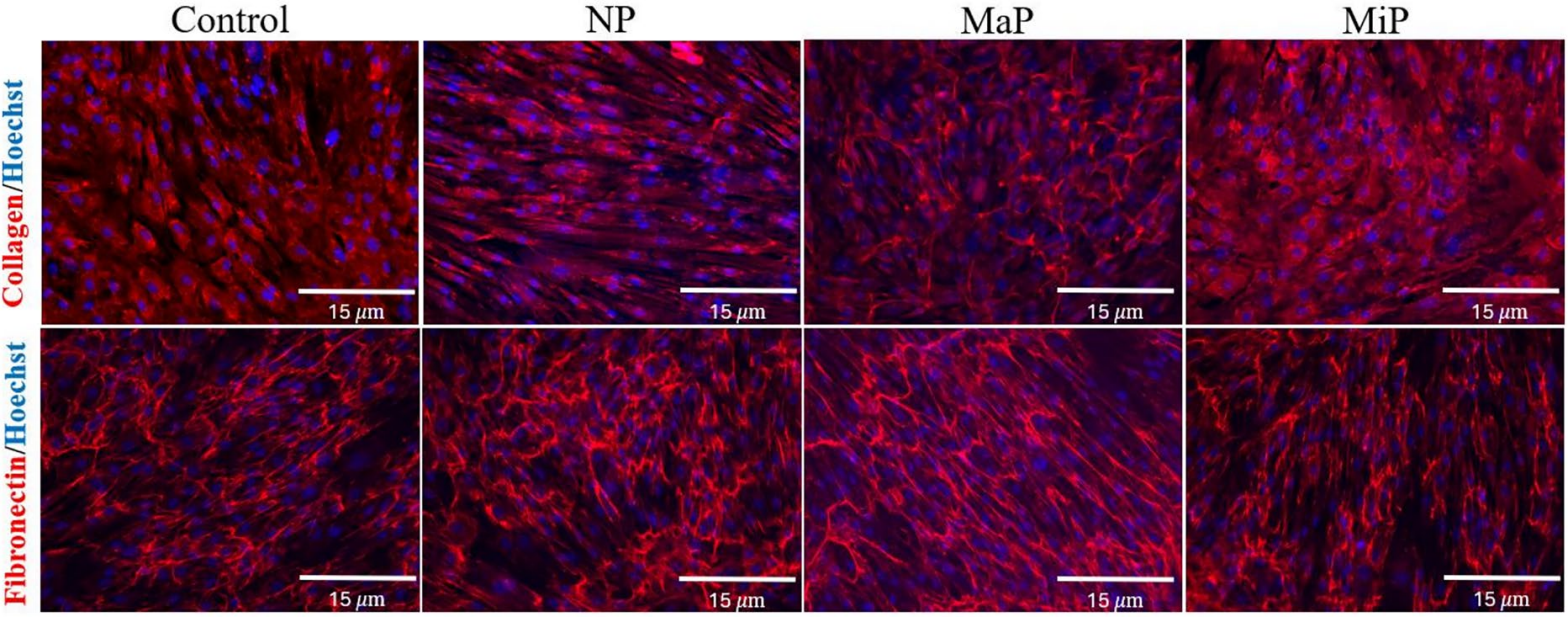
Immunocytochemistry of collagen type I (top row) and fibronectin (bottom row) after seven days of incubation with the titanium meshes with different surface modifications: Non-polished (NP), Matte-polished (MaP) and Mirror-polished (MiP) compared to control untreated cells. Nuclei were counterstained with Hoechst at Scalebar = 15$\mu m$.

**Fig. 5:**
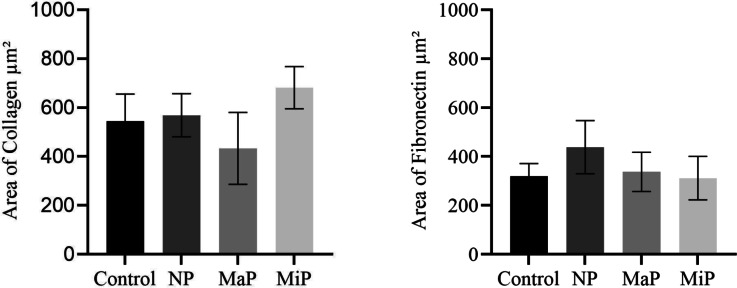
Area of the collagen and fibronectin in cells after seven days of incubation with the titanium meshes with different surface modifications: Non-polished (NP), Matte-polished (MaP) and Mirror-polished (MiP) compared to control untreated cells.

Image analysis was carried out on fibronectin- and collagen type I-stained NHDFs at confluency on day seven post-incubation with the various titanium meshes, and the results revealed that these cells were unaffected, as shown in Figs. 4 and 5 for collagen type I ($p> 0.2193$), and fibronectin ($p> 0.0507$). For example, NHDFs treated with the NP, MaP and MiP meshes presented areas positive for collagen type I at 568.86 ± 88.12 $\mu m^2$, 433.26 ± 147.02 $\mu m^2$, and 681.52 ± 86.14 $\mu m^2$, respectively, whereas untreated control cells were 544.54 ± 110.69 $\mu m^2$. Similarly, NHDFs treated with the NP, MaP and MiP meshes presented areas positive for fibronectin at 438.24 ± 109.05 $\mu m^2$, 336.97 ± 80.22 $\mu m^2$, and 311.62 ± 88.66 $\mu m^2$, respectively, whereas those of the untreated control cells were 318.82 ± 52.56 $\mu m^2$.

## Discussion

Titanium and its alloys are widely used for various applications in the implantable field. Previous research has extensively focused on examining the biocompatibility of titanium and its alloys on bone-anchored applications, such as dental implants and knee and hip prosthetics. However, little is known about the biocompatibility of titanium in soft-anchored or percutaneous implants, similar to our proposed implant for use in patients with a stoma.

In our present work, we 3D printed a titanium mesh implant and modified the surface topology into three distinctive surfaces of varying roughness, namely, NP (untreated surface) and two polished surfaces, via a tumbler machine with two different media, resulting in MaP and MiP surfaces. There were no significant differences observed in the cellular metabolic activity between NHDFs incubated with any of the titanium meshes and compared to the control cells incubated without any meshes.

The reduction of alamarBlue through living cells is proportional to the cell density. In our study, the three surfaces demonstrated increased metabolic activity over the culture period; no significant differences were observed between the groups. Naoki Y *et al.*^80^ examined cellular attachment on four different surface modifications, with different roughnesses. The results revealed that the sample roughness had no significant effect on the degree of cellular attachment. A similar trend was observed in a study conducted by Muataz O *et al.*^81^ when four different surface roughnesses were compared on two types of cell lines, namely, primary human gingival fibroblasts (HGFB) and keratinocytes (HGKC). In our proposed titanium implant construct, a 3D printed mesh structure was used, increasing the surface area with an aim for improved cellular adhesion without causing cytotoxic effects on various abdominal wall tissues. Interestingly, Carla H *et al.*^82^ compared the cellular behaviour on four different Ti-6Al-4V titanium alloy surfaces with varying surface roughnesses and reported that the porous (EBM) surface Ti-6Al-4V had increased cellular proliferation compared with the other groups. Together, our obtained data align with the previous studies, suggesting that surface modification of titanium mesh has no evident differences observed on cultured cells. Thus, our 2D in-vitro studies show cytocompatibility in the tested assays; however, further work using physiologically relevant 3D models and in-vivo studies is required before any clinical recommendations can be made.

Any implantable material must be both safe and biofunctional, causing no harm to the surrounding tissue. Although previous studies have shown that Ti-6Al-4V implants can undergo corrosion, releasing aluminium and vanadium ions that may induce cytotoxic effects or even contribute to Alzheimer’s disease^41^, we did not directly assess corrosion behaviour or ion release in this study. Instead, our conclusions are based on cell viability measurements, which showed that, across all three surface roughness variants of the titanium mesh, viability remained comparable to the control group. These findings indicate that, under the specific 2D NHDF culture conditions tested, neither the different surface finishes nor any potential ion release produced detectable cytotoxic effects. Within these limitations, the results support good cytocompatibility of the mesh with soft tissue surrounding the proposed stoma implant.

One of the fundamental effects of cell behaviour on the surface of an implant is the regeneration of soft tissue following the implantation, a process often associated with inflammation and wound healing^83,84^. To investigate this response, we evaluated extracellular matrix (ECM) protein levels, specifically collagen type I and fibronectin, in cells cultured in the presence or absence of titanium. These two proteins were selected due to their crucial roles in extracellular matrix remodelling and their association with fibrotic responses in soft tissue; elevated levels of these proteins are commonly observed in the early stages of fibrosis and may indicate abnormal wound healing or excessive tissue scarring around implants^85,86^. Collagen type I is essential for providing structural support to connective tissues and is important in repairing and healing tissues surrounding implants^87^. Fibronectin, an ECM glycoprotein, is important for cell adhesion and mediating cell-ECM interactions, facilitating adhesion, differentiation, and migration to the implant surface^88^. Our study did not reveal significant differences in the expression levels of these proteins on surfaces with varying roughness, suggesting that the presence of titanium does not adversely affect the production of these critical ECM components. Furthermore, the levels of collagen type I and fibronectin in our experimental groups were not markedly elevated compared to the control group, suggesting a minimal fibrotic response. These findings are indicative of the limited scar tissue formation. To the best of our knowledge, no prior study has specifically assessed the expression levels of collagen type I and fibronectin in the context of soft-anchored titanium implants.

This study has noteworthy limitations. All experiments were performed in 2D monolayer culture using a single primary cell type (NHDFs), which does not recapitulate the complex multicellular, three-dimensional architecture and mechanical environment of the abdominal wall. Furthermore, only short to mid-term time points (up to 22 days) were assessed, so long-term tissue responses, remodelling, and potential late-onset adverse effects were not evaluated. Finally, corrosion behaviour and ion release were not directly measured. As such, our conclusions are limited to short-term cytocompatibility outcomes in a simplified *in vitro* setting and should be interpreted accordingly. This needs to be addressed in future research.

In summary, our *in vitro* study demonstrated that variations in surface roughness of the Ti-6Al-4V mesh did not significantly affect NHDF metabolic activity, cytotoxicity, or ECM protein expression. These preliminary findings suggest that within the tested roughness range, surface modification may not be a critical factor for fibroblast cytocompatibility in simplified 2D culture conditions. However, this observation does not establish clinical readiness or eliminate the need for surface optimisation.

## Conclusion

In this study, we evaluated the effect of surface roughness of DMLS-fabricated Ti-6Al-4V mesh specimens (NP, MaP, and MiP) on NHDF metabolic activity, cytotoxicity, and ECM protein (collagen I and fibronectin) expression in a 2D in vitro model. Across the roughness spectrum tested, we did not detect statistically significant differences in these cytocompatibility endpoints, and none of the surfaces exhibited overt cytotoxicity. These findings suggest that, under simplified *in vitro* conditions, polishing of the titanium mesh may not be required to support fibroblast viability and ECM production. However, the results must be interpreted within the limitations of the model, which does not capture long-term, multicellular, mechanical, or infection-related aspects of soft tissue integration. Future work will include 3D co-culture systems, *in vivo* studies, and detailed corrosion and roughness characterisation to more comprehensively define the role of surface finish in the performance of soft anchored mesh implants for stoma applications.

## Methods

### Implant design and fabrication

Three implants composed of Ti-6Al-4V alloy were fabricated using direct metal laser sintering (DMLS) additive manufacturing via an EOS M280 400-W system with laser power of 280 *W*, scan speed of 1200 *mm*/*s*, hatch spacing of 0.14 *mm*, and layer thickness of 30 $\mu$*m* using an argon atmosphere and recycled powder within the manufacturer’s specifications (Protolabs). The mesh structure featured an inner diameter of approximately 12 *mm*, with a broader attachment ring measuring approximately 50 *mm* in diameter for integration with a flexible valve. The mesh pores had a diamond-like shape with a mean height of 1.66 ± 0.02 *mm* and a mean width of 1.88 ± 0.05 *mm*, measured from optical microscope images (Olympus BX53M with SC50 camera) at three locations per direction, and the corresponding strut thickness was approximately 1.51 ± 0.04 *mm*.

**Fig. 6:**
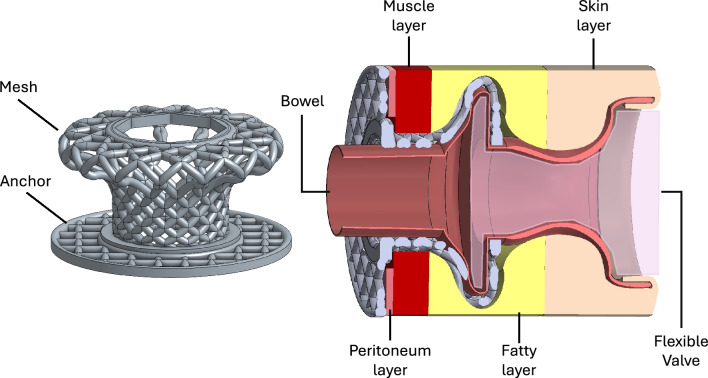
Schematic representation of the Ti-6Al-4V mesh implant and its intended surgical placement for stoma management. (left) Illustration of the mesh with an integrated anchor; (right) the implant positioned within the abdominal wall, with the anchor seated posterior to the peritoneum and secured with sutures, the mesh traversing the muscular layer and residing within the subcutaneous fat, and the distal flexible valve fixed within the mesh and extending to the skin surface, where it functions as a controllable outlet to regulate waste excretion.

Micro-CT scanning ($\mu$CT50; Scanco Medical, Bassersdorf, Switzerland) was performed to further characterise the mesh structure. Three-dimensional images were analysed using Dragonfly software (Dragonfly 3D Visualisation and Analysis Software, v2025.1, https://www.theobjects.com/dragonfly/). Image analysis employed automatic segmentation via upper Otsu thresholding for the titanium phase and lower Otsu thresholding for the pore space. The multi-ROI command function was utilised to isolate and characterise the interconnected pore network. Volumetric porosity was calculated as the ratio of pore volume to total implant volume, which was determined to be 0.20%. The mesh architecture was specifically engineered to facilitate tissue ingrowth and enhance fixation to the surrounding biological tissue, as illustrated in the Fig. 6. The implant’s basal anchoring structure was designed for suturing to the peritoneum, thereby enabling bowel passage while minimising the risk of parastomal hernia.

### Surface modification of the titanium implant

**Fig. 7:**
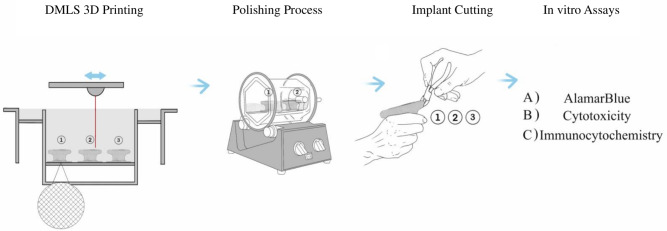
Study design illustrating strategy of DMLS 3D printing of three implants depicted as (1), (2), and (3), followed by surface modification of implant (1) and (2) using tumbler machine producing (1) Matte polished surface (MaP) and (2) Mirror polished surface (MiP), while implant (3), non-polished surface (NP) is kept in its raw and rough surface, then cutting all three implants into smaller specimen pieces for in-vitro analysis using Alamar blue, cytotoxicity, and immunocytochemistry.

The first implant was left with its natural coarse surface, non-polished (NP), whereas the other two implants underwent physical alteration on their surfaces through an outsourcing polishing process from UK Metal Polishers LTD. Both altered implants were polished via a three-phase rotary polishing process (UK Metal Polishers Ltd.). In the first phase, a coarse ceramic media was used for approximately 2–3 hours to reduce the largest asperities. This was followed by an intermediate plastic media for 2 hours to further smooth the surface, and a final fine media with polishing compound for 2 hours to achieve the matte (MaP) and mirror (MiP) finishes. All three implants were then cut manually into 10 small specimens each, with each specimen varying in both shape and size, with an average surface area of 45.51 ± 4.82 $mm^2$. The fabrication and surface modification processes are summarised in Fig. 7.

### Surface morphology characterisation of the Ti-6Al-4V

The samples from each implant were ultrasonically washed and cleaned separately and sequentially with 100% ethanol, 100% acetone and distilled water for five minutes each. To characterise the surfaces of the samples, a 4 K ultra-high accuracy VHX-7000N digital microscope with a VHX-Z20 lens (KEYENCE (UK) Ltd.) was used. Moreover, surface roughness analysis was performed via the same microscope after an area was chosen from the 3D display. For roughness analysis, three randomly selected fields per surface type were analysed using the 3D profile mod, and the Ra was extracted for each field and averaged.

### Sample preparation and sterilisation

After microscopy images were obtained, the samples were then washed again sequentially with ethanol, acetone and distilled water for five minutes each and sterilised by autoclaving at 132$^{\circ }$C for a 20-minute cycle.

### Cell culture

Adult normal human dermal fibroblasts (NHDFs), purchased from Sigma-Aldrich (Sigma-Aldrich, UK), were cultured in flasks (Thermo Fisher Scientific, UK) with prewarmed fibroblast growth medium 2 media containing basal media and 5% SupplemenMix (PromoCell, UK) and incubated at 37$^{\circ }$C with 5% $CO_2$. The medium was changed every three days until the cells reached 90% confluence. The cells were then passaged via a DetachKit (Sigma-Aldrich) containing wash buffer, trypsin, and neutralising buffer^89^.

### Cell viability assay

An alamarBlue colourimetric assay (Fisher Scientific, UK) was used to assess cell metabolic activity via the suspension seeding culture method^68^. NHDFs were plated at $4 \times 10^3$ cells/mL in a 24-well plate with and without the respective titanium samples for the duration of the study and incubated under standard culture conditions. The percentage viability was measured over 22 days using Equation 1:

1 $$\begin{aligned} \begin{aligned} \text {Percentage viability} = \frac{{O_{\text {2}} \times A_{1} - O_{\text {1}}}\times A_{2}}{{O_{\text {2}} \times P_{1} - O_{\text {1}}} \times P_{2}} \times 100\% \end{aligned} \end{aligned}$$

where $O_1$ is the molar extinction coefficient (E) of oxidised alamarBlue at 570 nm, $O_2$ is the coefficient (E) of oxidised alamarBlue at 600 nm, $A_1$ is the absorbance of test samples at 570 nm, $A_2$ is the absorbance of test wells at 600 nm, $P_1$ is the absorbance of control at 570 nm, and $P_2$ is the absorbance of control at 600 nm.

### Cytotoxicity MTS assay

The cytotoxicity of the titanium samples was evaluated via a colourimetric 3-(4,5-dimethylthiazol-2-yl)−5-(3 carboxymethoxyphenyl)2-(4 sulfophenyl)−2H-tetrazolium (MTS) assay (Promega, UK). The cells were seeded at $4 \times 10^4$ cells/mL per well in a 24-well plate with the respective samples for the duration of the study and incubated under standard culture conditions. On day five, a 1% volume of MTS reagent was added to the respective wells and incubated for four hours at 37$^{\circ }$C with 5% $CO_2$. The absorbance of MTS was measured at 490 nm via a SPECTROStar Nano microtiter plate reader (BMG LABTECH, Ortenberg, Germany), which was used to determine the amount of formazan product present in each well. Cell viability was calculated based on the absorbance of the respective wells corrected with blank subtraction via Equation 2:

2 $$\begin{aligned} \begin{aligned} \text {Absorbance} ={{Absorbance_{\text {test}} - Absorbance_{\text {blank}}}} \end{aligned} \end{aligned}$$

### Fibronectin and collagen immunocytochemistry

Fibroblasts ($4 \times 10^4$ cells/mL) were plated in a 24-well plate with the respective implants and cultured until they reached confluence (approximately seven days). The cells were then washed with PBS and fixed in 4% paraformaldehyde for 10 minutes, followed by permeabilisation with 0.1% Triton-X 100 for 10 minutes on ice. The fibroblasts were then incubated in 1% BSA in PBS for 30 minutes at room temperature, followed by overnight incubation at 4 °C with the following primary antibodies: a rabbit polyclonal anti-fibronectin antibody (Antibodies, Cambridge, UK; 1:200 dilution) or a rabbit polyclonal anti-collagen type I antibody (Antibodies, Cambridge, UK; 1:200 dilution). The cells were washed with wash buffer containing PBS and 0.01% Tween-20 and incubated with the secondary antibody Alexa 555-conjugated goat anti-rabbit IgG (1:200 dilution) for approximately one hour, followed by Hoechst (1:1000 dilution) for 10 minutes at room temperature. Fluorescence images were captured on an Invitrogen EVOS M5000 (Thermo Fisher Scientific, UK) fluorescence microscope. Five fields were captured for each group at 20x magnification and a scale bar of 150 $\mu \text {m}$ under fixed voltage intensity. Using ImageJ, the area of fluorescence intensity was measured and normalised against the number of nuclei.

### Statistical analysis

The data were plotted as means +/- standard deviations and analysed via GraphPad Prism (GraphPad Prism v10.3.1, https://www.graphpad.com). Two-way ANOVA with a Tukey correction test were used to determine statistical significance for the alamarBlue and MTS assays. One-way ANOVA was used to analyse the quantified immunocytochemistry images. A value of $p < 0.05$ was considered statistically significant.

## Data Availability

The datasets used and/or analysed during the current study available from the corresponding author on reasonable request.
